# Lung tumorspheres reveal cancer stem cell-like properties and a score with prognostic impact in resected non-small-cell lung cancer

**DOI:** 10.1038/s41419-019-1898-1

**Published:** 2019-09-10

**Authors:** Alejandro Herreros-Pomares, Juan Diego de-Maya-Girones, Silvia Calabuig-Fariñas, Rut Lucas, Alicia Martínez, José Miguel Pardo-Sánchez, Sergio Alonso, Ana Blasco, Ricardo Guijarro, Miguel Martorell, Eva Escorihuela, María Dolores Chiara, Elena Duréndez, Carolina Gandía, Jerónimo Forteza, Rafael Sirera, Eloísa Jantus-Lewintre, Rosa Farràs, Carlos Camps

**Affiliations:** 1 Molecular Oncology Laboratory, Fundación Hospital General Universitario de Valencia, Valencia, Spain; 2 CIBERONC, Valencia, Spain; 3 Oncogenic Signalling Laboratory, Centro de Investigación Príncipe Felipe, Valencia, Spain; 4 Department of Pathology, Universitat de València, Valencia, Spain; 5 Department of History of Science and Documentation, Universitat de València, Valencia, Spain; 6 Cytomic Core Facility, Centro de Investigación Príncipe Felipe, Valencia, Spain; 7 Program of Predictive and Personalized Medicine of Cancer, Institut de Reserca Germans Trias i Pujol (PMPPC-IGTP), Badalona, Spain; 8 Department of Medical Oncology, Hospital General Universitario de Valencia, Valencia, Spain; 9 Department of Thoracic Surgery, Hospital General Universitario de Valencia, Valencia, Spain; 10 Department of Pathology, Hospital General Universitario de Valencia, Valencia, Spain; 11 Institute of Sanitary Research of Asturias, Hospital Central de Asturias, Universidad de Oviedo, Oviedo, Spain; 12 Instituto Valenciano de Patología, Unidad Mixta de Patología Molecular, Centro Investigación Príncipe Felipe/Universidad Católica de Valencia, Valencia, Spain; 13 Department of Biotechnology, Universidad Politécnica de Valencia, Valencia, Spain; 14 Department of Medicine, Hospital General Universitario de Valencia, Valencia, Spain

**Keywords:** Non-small-cell lung cancer, Cancer stem cells, Tumour biomarkers, Stem-cell research

## Abstract

The high resistance against current therapies found in non-small-cell lung cancer (NSCLC) has been associated to cancer stem-like cells (CSCs), a population for which the identification of targets and biomarkers is still under development. In this study, primary cultures from early-stage NSCLC patients were established, using sphere-forming assays for CSC enrichment and adherent conditions for the control counterparts. Patient-derived tumorspheres showed self-renewal and unlimited exponential growth potentials, resistance against chemotherapeutic agents, invasion and differentiation capacities in vitro, and superior tumorigenic potential in vivo. Using quantitative PCR, gene expression profiles were analyzed and *NANOG*, *NOTCH3*, *CD44*, *CDKN1A*, *SNAI1*, and *ITGA6* were selected to distinguish tumorspheres from adherent cells. Immunoblot and immunofluorescence analyses confirmed that proteins encoded by these genes were consistently increased in tumorspheres from adenocarcinoma patients and showed differential localization and expression patterns. The prognostic role of genes significantly overexpressed in tumorspheres was evaluated in a NSCLC cohort (*N* = 661) from The Cancer Genome Atlas. Based on a Cox regression analysis, *CDKN1A*, *SNAI1*, and *ITGA6* were found to be associated with prognosis and used to calculate a gene expression score, named CSC score. Kaplan–Meier survival analysis showed that patients with high CSC score have shorter overall survival (OS) in the entire cohort [37.7 vs. 60.4 months (mo), *p* *=* 0.001] and the adenocarcinoma subcohort [36.6 vs. 53.5 mo, *p* = 0.003], but not in the squamous cell carcinoma one. Multivariate analysis indicated that this gene expression score is an independent biomarker of prognosis for OS in both the entire cohort [hazard ratio (HR): 1.498; 95% confidence interval (CI), 1.167–1.922; *p* = 0.001] and the adenocarcinoma subcohort [HR: 1.869; 95% CI, 1.275–2.738; *p* = 0.001]. This score was also analyzed in an independent cohort of 114 adenocarcinoma patients, confirming its prognostic value [42.90 vs. not reached (NR) mo, *p* = 0.020]. In conclusion, our findings provide relevant prognostic information for lung adenocarcinoma patients and the basis for developing novel therapies. Further studies are required to identify suitable markers and targets for lung squamous cell carcinoma patients.

## Introduction

Lung cancer is the most commonly diagnosed cancer and the leading cause of cancer death worldwide, with ~15% of patients surviving 5 years after diagnosis^1^. Eighty-five percent of diagnosed patients are classified as non-small-cell lung cancer (NSCLC), which includes adenocarcinoma (ADC), squamous cell carcinoma (SCC), and large-cell carcinoma, and 75% are diagnosed at advanced stages, when surgery is not possible^2^. Significant advances in the development of treatments against driver mutations and immune-based therapies for these patients were achieved in recent years^3,4^, but many patients still develop treatment resistance, progress, and die^5,6^. Curative surgery is the standard of care for early-stage patients with a good performance status, but the recurrence rate ranges from 35 to 50% and, after an apparently successful surgical treatment, appearance of secondary tumors often leads to the relapse of resected patients^7^. This poor prognosis greatly supports the efforts to establish prognostic biomarkers and therapeutic targets for improving the management of NSCLC.

Among these targets, cancer stem-like cells (CSCs) are proposed as a promising tumor population, since they are believed to survive after conventional cancer treatments and regenerate tumors even when are undetectable^8^. These slow-diving cells are characterized by their self-renewal potential, the capacity to undergo asymmetric division to highly proliferative cells, and a great tumorigenic activity, acting like tumor-initiating cells and producing aggressive tumors when transplanted in immune-compromised mice^9^. Additionally, CSCs share with other stem cells the overexpression of cytoprotective enzymes, enhanced ability to efflux molecules, and the anchorage-independent growth ability, which can be used for enriching cell cultures in this population. Nevertheless, specific strategies against CSCs are not approved in clinical practice and a better understanding of their impact on patients’ prognosis is required.

CSCs have been analyzed in several solid tumors, including brain, lung, breast, colon, or pancreas, finding aberrant expression of different molecules and signaling pathways^10–14^. However, most of the studies have been performed on cell lines and conflicting data can be found. For instance, CD133 and CD44 molecules have been successfully used to identify lung cancer cells with CSC properties in some studies^15,16^, whereas other publications reported that CD133^−^ or CD44^−^ cell populations also possess the ability for self-renewal and enhanced tumor initiation capacity when transplanted into mice^17,18^. Cell lines are a powerful tool and offer several advantages over primary cells, but they do not completely mimic them and studies including primary cultures are required when analyzing stemness properties, markers, pathways, and novel approaches. To date, few publications include primary cultures from NSCLC patients’ tissue and, if included, the number of patients is small, since primary culture establishment is difficult and CSCs constitute an uncommon population^19–21^.

The aim of this study was to characterize the population of CSCs derived from resected NSCLC patients to identify genes and molecules that could have a prognostic role or constitute the basis for developing novel therapies focusing on this tumor population. We have confirmed that tumorspheres from NSCLC patients are enriched in cells with stem properties, identified potential targets against this aggressive population, and established a three-gene signature that is an independent prognostic marker for patients’ OS.

## Materials and methods

### Patients and tissue samples

This study included 134 patients from the General University Hospital of Valencia who underwent surgery between 2004 and 2016 and who fit the eligibility criteria: resected, non-pretreated stage I–IIIA patients (according to the American Joint Committee on Cancer staging manual) with a histological diagnosis of NSCLC. Lung tumor specimens were obtained at the time of surgery. Tumor samples from 20 patients were immediately processed for primary culture establishment. The rest of samples were preserved in RNAlater (Applied Biosystems, USA) to avoid degradation of RNA and were frozen at −80 °C until gene expression analyses. The mutation status of *KRAS*, *TP53*, *EGFR*, A*LK*, and *ROS1* was assessed for the whole cohort. *KRAS* gene mutations in codons 12, 13, and 61 were quantitatively detected by pyrosequencing using the theraScreen® KRAS Pyro® kit (Qiagen, Germany). *EGFR* mutations were analyzed by quantitative real-time PCR (RTqPCR) using the theraScreen® EGFR RGQ PCR (Qiagen, Germany), whereas *TP53* mutations were determined using standard PCR followed by Sanger sequencing. ALK and ROS1 rearrangements were determined by immunohistochemistry (IHC) using ALKp80 (MAD-001720QD) and ROS1 (MAD-000746QD). Antibodies were from Master Diagnostica (Granada, Spain), respectively.

### Establishment of primary cell cultures

Unless specified, all reagents were obtained from Gibco Paisley, UK. Surgical tumor specimens were washed and minced into small pieces. Tumor dissociation was carried out by enzymatic digestion (1 mg/mL collagenase type IV, 1 mg/mL dispase, and 0.001% DNAse, Sigma, St. Louis, USA) for 3 h at 37 °C. Half of cells were cultured in collagen-coated flasks with Advanced DMEM-F12 supplemented with 10% fetal bovine serum (FBS), 200 µg/mL penicillin/streptomycin, and 2 mM l-glutamine. The rest of the cells were seeded at low density in ultra-low attachment plates (Corning, Lowell, MA, USA) with serum-free Advanced DMEM-F12 medium supplemented with 0.4% bovine serum albumin (BSA), 50 µg/mL epidermal growth factor (EGF), 20 µg/mL basic fibroblast growth factor (bFGF), 5 µg/mL insulin-transferrin-selenium (ITS) PREMIX (Corning, Lowell, MA, USA), 2% B-27, 200 µg/mL penicillin/streptomycin, and 2 mM l-glutamine to support their growth as undifferentiated tumorspheres. Cultures were expanded by mechanical dissociation of spheres, followed by re-plating of both single cells and residual small aggregates in complete fresh medium. In all cases, cells were maintained at 37 °C in 5% CO_2_ atmosphere and the medium was replaced twice a week.

### Cell line cultures

A549, NCI-H1395, NCI-H1650, NCI-H1975, NCI-H1993, NCI-H2228, NCI-H23, NCI-H358, NCI-H460, HCC827, PC9, and SW900 cells were purchased from American Type Culture Collection (Supplementary Table S1). Cell lines were cultured in RPMI-1640 containing 10% FBS, 200 µg/mL penicillin/streptomycin, and 0.001% non-essential amino acids. To obtain tumorspheres, the cells were trypsinized using 0.05% trypsin-EDTA when they reached 80% confluence. The cells were seeded at low density in ultra-low attachment flasks with serum-free RPMI-1640 medium supplemented with 0.4% BSA, 50 µg/mL EGF, 20 µg/mL bFGF, 5 µg/mL ITS PREMIX, 2% B-27, 200 µg/mL penicillin/streptomycin, and 2 mM l-glutamine.

### Animals and xenografts

To test the tumorigenic potential of adherent cells and tumorspheres, 6-week-old NOD.CB17-Prkdc^scid^/NcrCrl mice (Jackson Laboratories) were subcutaneously transplanted with cell suspensions in serum-free medium and Matrigel (BD) (1:1). Tumor volume (TV) measurements were recorded once a week using the formula: TV (mm^3^) = *d*^2^ × *D*/2, where *d* and *D* are the shortest and the longest diameter, respectively^22^. Animals were terminated when xenografts were 1000 mm^3^.

### Cell invasion assays and time-lapse video recording

For cell invasion assays, cells were cultured in the medium used for tumorsphere formation supplemented with 0.2% methylcellulose in a non-adhesive convex environment for 12 h at 37 °C and 5% CO_2_. Tumorspheres were mixed with collagen matrix (2.5 mg/ml) and incubated for 30 min at 37 °C prior to microscopic analysis. Time-lapse microscopy imaging was performed on a Zeiss AxioObserver Z1 microscope with a Plan-Apochromat ×40 /1.3 (NA = 1.3, working distance = 0.21 mm), a camera, and an Apotome attachment (Carl Zeiss, Germany). Mosaic images were collected using AxioVision software over a period of 20 h with a time resolution of 30 min.

### Cell growth curves

Cultures were trypsinized at 80% confluence and counted in a Neubauer camera with Trypan Blue dye exclusion (Sigma, USA). Tumor cells were plated at a density of 1000 cells per well in 96-well plates and cell viability was evaluated 24, 48, and 72 h after seeding with the CellTiter 96^®^ Aqueous One Solution Cell Proliferation Assay (Promega, WI, USA) according to standard protocols and analyzed with a Victor 3 plate reader (Wallac, Turku, Finland). Data represented are the mean of three replicates in three independent experiments.

### Cytotoxicity assays

Adherent cells and lung tumorspheres were cultured at desired density according to their growth curves into 96-well plates. Chemotherapeutic agents were added after 24 h at the following final concentrations: cisplatin 50 µM, docetaxel 10 µM, paclitaxel 10 µM, vinorelbine 10 µM, and pemetrexed 50 µM (Selleckchem, Germany). The selective agent against CSCs, salinomycin, was added at 1 µM (Selleckchem, Germany). Cells treated with dimethyl sulfoxide (vehicle control) served as controls. Cell viability was evaluated after 48 h with the CellTiter 96^®^ Aqueous One Solution Cell Proliferation Assay and analyzed with a Victor 3 plate reader. Data represented are the mean of three replicates in three independent experiments.

### Gene expression analysis

RTqPCR was performed to analyze the relative expression of 51 CSC-related genes on a Roche LightCycler^®^480 II system (Roche Ltd., Basel, Switzerland) (Supplementary Table S2). RNA from cell pellets and frozen tissue samples was extracted using standard TRIZOL (Invitrogen) method. Reverse transcription reactions were performed from 1.0 µg of total RNA using random hexanucleotides and a High-Capacity cDNA (complementary DNA) Reverse Transcription Kit (Applied Biosystems, USA) following the manufacturer’s instructions. The thermal cycling conditions were as follows: 10 min at 25 °C, 120 min at 37 °C, and 5 s at 85 °C. RTqPCR was performed with assays based on hydrolysis probes using 1 µL of cDNA, TaqMan Gene Expression Master Mix, and a TaqMan Gene Expression Assay (Applied Biosystems, USA) in a 5 µL final reaction volume. The thermal cycling parameters were as follows: 2 min at 50 °C and 10 min at 95 °C, followed by 40 cycles of 15 s at 95 °C and 1 min at 60 °C. For efficiency calculations, we used random-primed qPCR Human Reference cDNA (Clontech, USA). *ACTB*, *GUSB*, and *CDKN1B* were selected as endogenous controls using GeNorm software. Relative gene expression levels were expressed as the ratio of target gene expression to the geometric mean of the endogenous gene expressions according to Pfaffl formula^23^.

### Immunoblot analysis

Tumorspheres were washed with cold phosphate-buffered saline (PBS) and lysed on ice with lysis buffer (50 mM Tris-HCl, pH 7.5, 150 mM NaCl, 0.02% NaN_3_, 0.1% SDS, 1% NP40, 0.5% sodium deoxycholate, 2 mg/ml leupeptin, 2 mg/ml aprotinin, and 1 mM phenylmethylsulfonyl fluoride), whereas adherent cells were washed with cold PBS and scraped out of the dishes before lysis. Proteins were separated by sodium dodecyl sulfate-polyacrylamide gel electrophoresis, transferred to polyvinylidene difluoride membranes, probed with the indicated antibodies (Supplementary Table S3), and detected by chemiluminescence. Densitometric analysis was performed using ImageJ (NIH, USA) and all results were normalized over β-actin.

### Immunofluorescence analysis

Cells were fixed in 4% paraformaldehyde in PBS at room temperature for 15 min, washed and permeabilized with 0.4% Triton X-100 in PBS for 10 min, and washed again. Fixed cells were blocked in PBS containing 10% BSA and 0.4% Triton X-100 for 1 h. Immunodetection was carried out using the same antibodies described above (Supplementary Table S3). Cells were incubated overnight at 4 °C with primary antibodies in blocking buffer. Thereafter, secondary antibodies contained in the blocking buffer were incubated for 1 h. Slides were incubated with 4′,6-diamidino-2-phenylindole for 3 min, mounted with Fluoromount Aqueous Mounting Medium (Sigma, USA), and analyzed using a Leica confocal microscope (Leica Microsystems, IL, USA).

### IHC analysis

Adherent cells and tumorspheres were fixed in 4% paraformaldehyde in PBS at room temperature for 15 min, washed, and embedded in Richard-Allan Scientific™ HistoGel (Thermo Scientific, UK) prior to paraffin. Immunodetection was done using CD56 (MAD-000749QD), CEAm (MAD-002095QD), CEAp (MAD-001115QD), cytokeratin 5/6 (MAD-000651QD), cytokeratin 7 (MAD-001004QD), and p63 (MAD-000479QD) antibodies from Master Diagnostica (Granada, Spain).

### Bioinformatic analysis

In silico analysis was performed using two lung cancer data sets from the The Cancer Genome Atlas (TCGA) consortium^24,25^. Clinical and RNA-sequencing (Illumina HiSeq platform) information was directly downloaded from the ICGC Data Portal^26^, https://dcc.icgc.org/releases/current/projects/LUAD-US, and https://dcc.icgc.org/releases/current/projects/LUSC-US.

### Statistical analysis

Expression of paired adherent cells and tumorspheres were analyzed using non-parametric Wilcoxon’s signed-rank test. In order to reduce the dimensionality and remove possible collinear expression of genes, a logistic regression model was built using a stepwise selection and minimizing Akaike’s information criterion to select the genes, which contributed more to differentiate tumorspheres from adherent cells. Continuous variables were compared by non-parametric Mann–Whitney *U* and Kruskal–Wallis tests. A Spearman’s rank test was used to test for correlations between continuous variables and the association between discrete variables was evaluated by the *χ*^2^ test. Survival analyses were performed using univariate Cox regression analysis and Kaplan–Meier (log-rank) test method with clinicopathological variables and dichotomized gene expression levels. To assess the independent value of the tested biomarkers, a Cox proportional hazard model for multivariate analyses was used. All significant variables from the univariate were entered into the multivariate analyses in a forward stepwise Cox regression analysis. Furthermore, we also calculated gene expression score based on multi-gene signature using a method previously reported^27,28^. Univariate Cox regression analysis was used to select genes associated with mortality (*Z*-score >1.5), which were afterwards included in a multivariate risk model. All genes were included for these purposes, and expression values for all analyses were continuous variables. A probability of 95% (*p* < 0.05) was considered statistically significant for all analyses. Statistical analyses were performed using the Statistical Package for the Social Sciences (SPSS, Chicago, IL, USA) version 15.0. Principal component analysis (PCA) for gene expression analyses were performed with the SIMCA-P software (version 13.0, Umetrics, Umea, Sweden) using unit variance scaling method.

## Results

### Generation of adherent and non-adherent primary NSCLC cultures

Clinicopathological information from each of the 20 patients included in this part of the study is summarized in Table 1. The median patient age was 68 years [range: 54–83], 65% were males, 65% had ADC, and 55% of patients were diagnosed at stage I of the disease. The median follow-up was 17.32 months [range: 5.1–33.23], and eight (40%) relapsed or died during the follow-up period.

**Table 1:** Clinicopathological characteristics and lung tumorspheres formation of the patients included in the study^a^

Patient code	Gender	Age (years)	TNM stage	Histology	Smoking status	Progression/exitus	DFS (months)	Mutational status	Tumorspheres formation
FIS291	Male	66	IIA (T2aN0M0)	SCC	Current	Yes	25.77	*KRAS* G12C	No
FIS299	Male	69	IIIA (T4N1M0)	SCC	Former	Yes	6.10	*TP53* K132E	Yes
FIS301	Male	71	IIB (T3N0M0)	SCC	Former	No	30.77	*TP53* D259fs*84	Yes
FIS302	Female	74	IIA (T2aN0M0)	ADC	Never	No	33.23	*KRAS* G12D*, TP53* E285K	Yes
FIS303	Female	57	IB (T2aN0M0)	ADC	Former	Yes	11.13	*TP53* R175H	Yes
FIS308	Male	72	IIB (T3N0M0)	SCC	Current	No	27.80	No mutation detected	No
FIS310	Male	68	IIIA (T3N2M0)	ADC	Former	Yes	6.43	*TP53* V157F R213SNP	No
FIS312	Male	62	IA (T1bN0M0)	ADC	Current	No	24.80	No mutation detected	No
FIS315	Female	65	IA (T1aN0M0)	ADC	Never	No	20.93	No mutation detected	Yes
FIS317	Male	76	IIB (T3N0M0)	SCC	Current	Yes	18.40	No mutation detected	Yes
FIS320	Male	65	IB (T2aN0M0)	ADC	Current	No	23.60	*TP53* P153fs*26	Yes
FIS321	Male	83	IB (T2aN0M0)	SCC	Current	No	22.50	*TP53* Q156*	No
FIS325	Female	67	IB (T2aN0M0)	ADC	Never	No	16.97	*EGFR* L858R	No
FIS326	Female	64	IB (T2aN0M0)	ADC	Former	Yes	6.97	*EGFR* L858R, *TP53* G244C	No
FIS330	Male	54	IA (T1aN0M0)	ADC	Current	No	5.10	*TP53* R283P	No
FIS331	Male	75	IIA (T2aN1M0)	ADC	Current	Yes	6.20	*TP53* R175H	No
FIS337	Male	73	IB (T2aN0M0)	ADC	Former	No	8.27	*KRAS* G12S	No
FIS343	Female	60	IB (T2aN0M0)	ADC	Former	Yes	7.00	*TP53* R158L	Yes
FIS345	Male	74	IIIA (T1aN2M0)	SCC	Current	No	7.80	No mutation detected	No
FIS347	Female	68	IB (T2aN0M0)	ADC	Never	No	17.67	No mutation detected	No

*DFS* disease-free survival, *ADC* adenocarcinoma, *SCC* squamous cell carcinoma, *WT* wild type

^a^No significant associations were found between clinicopathological characteristics and tumorspheres formation

**Fig. 1:**
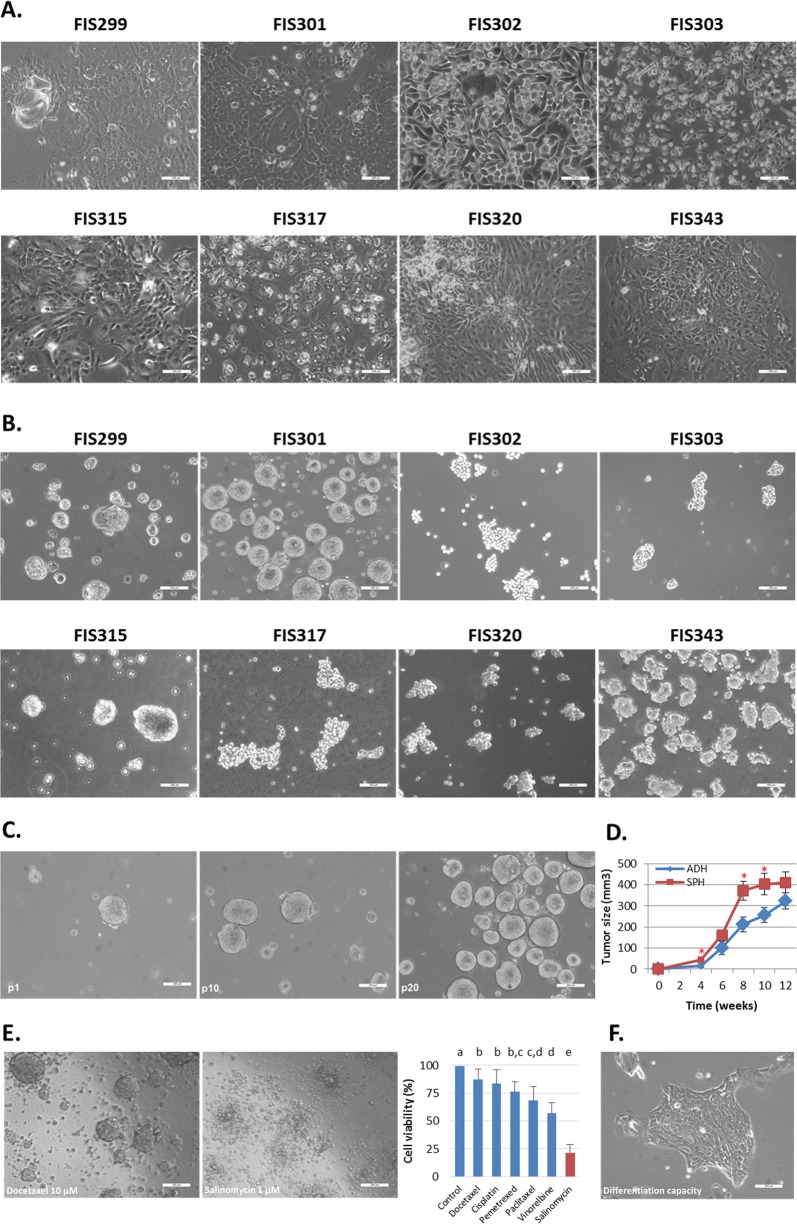
Results for the primary cultures establishment and characterization. **a** Representative images of the primary patient-derived cancer cells grown under adherent conditions. **b** Representative images of cells from the same patients under suspension conditions. **c** Self-renewal and unlimited exponential growth potentials of suspension cultures. Tumorspheres displayed stable growth without declining in number. **d** Tumor development capacity of adherent cells (blue line) and tumorspheres (red line) in vivo. The graph shows tumor growth in mice after injection of tumorspheres and their adherent counterparts at the indicated time points. Error bars represent SEM. *P < 0.01. **e** Representative images of the cytotoxic effects of the exposure of FIS301 patient tumorspheres to chemotherapeutic agents and salinomycin. Bar chart represents the cell viability of tumorspheres from primary cultures after 48 h exposure to cisplatin 50 µM, docetaxel 10 µM, paclitaxel 10 µM, vinorelbine 10 µM, pemetrexed 50 µM, and salinomycin 1 µM. ^a,b,c,d^Bars with different superscripts are statistically different (*p* < 0.05). **f** Differentiation capacity of lung tumorspheres. Under adherent conditions, tumorspheres adhere and acquire the morphologic features of cells directly established from tumor tissue

Primary patient-derived lung cancer cell cultures were maintained for 4 weeks before they were split for the first passage. Patient-derived cultures were successfully established in 8 out of 20 cases (40%), being able to grow tumor cells as monolayers and tumorspheres. No significant associations were found between the analyzed clinicopathological variables and the establishment of primary cultures. The morphology of cells from patient-derived cultures was examined and heterogeneity was observed on the adherent-cultured cells between samples (Fig. 1a). Cells from patients FIS299 and FIS301 grew as multilayers and formed cell colonies. These cell cultures showed abundant cell–cell interactions in the form of filopodia and lamellipodia and presence of giant cells and vesicles (Supplementary Fig. S1). FIS317 cells were cubic, grew as a monolayer, but showed tight cell–cell contact with filopodia and a high number of vesicles. In contrast, cells from patients FIS302, FIS303, and FIS315 were more elongated, with brighter nuclei, fewer interactions, and a more isolated growth. FIS320 and FIS343 cells were similar to patients FIS302, FIS303, and FIS315 in terms of growth rate (Supplementary Fig. S2), but had a different morphology. Regarding tumorspheres, tight spheroids were formed by FIS299, FIS301, and FIS315 cultures, whereas FIS302, FIS303, FIS317, and FIS320 formed more loose and irregularly shaped spheres, and FIS343 showed a mixed behavior (Fig. 1b). Simultaneously, cell line cultures were established in both conditions, adherence and suspension, and were included in further gene expression analyses (Supplementary Fig. S3).

### Lung tumorspheres exhibit stemness features

One of our aims was to analyze if lung tumorspheres displayed stemness properties. To determine the self-renewal and growth potentials of lung tumorspheres, we maintained the suspension cultures for more than 6 months. In all cases, lung tumorspheres exhibited stable unlimited exponential growth even in later passages (>30 passages) (Fig. 1c). To determine the invasive and tumorigenic capacities of tumorspheres, time-lapse video microscopy was performed, revealing that tumorspheres possess a high invasive capacity, being able to migrate through collagen matrix (Supplementary Video 1). We also evaluated the ability of tumorspheres and its corresponding adherent counterparts to develop tumors in vivo by subcutaneous transplantation of cells into immunocompromised mice. Both cells derived from tumorspheres and adherent cultures were able to initiate tumors in vivo, being the tumor latency higher in tumors induced by adherent cells (Fig. 1d). It is characteristic of CSCs to be highly resistant to conventional therapies as well. We investigated the cytotoxic activity of cisplatin, paclitaxel, vinorelbine, and pemetrexed at high doses. A selective agent against stem cells, salinomycin, was also tested at lower concentration. All chemotherapeutic drugs had a mild effect on tumorspheres from primary cultures after 48 h of exposure to antineoplastic agents, not reaching the half-maximal inhibitory concentration (Fig. 1e). Cisplatin, docetaxel, and pemetrexed displayed a modest cytotoxic effect with 83.5%, 86.9%, and 76.2% of cells alive after treatment, respectively. Paclitaxel and vinorelbine were more effective with 68.2% and 56.9% of cells alive after exposure, respectively. In contrast, salinomycin showed higher cytotoxic activity against tumorspheres with 21.7% of cells alive after 48 h exposure. Moreover, these drugs were more effective in cells cultured under adherent conditions (Supplementary Fig. S4). Finally, we assessed the differentiation potential of tumorspheres. Using serum-containing medium and conventional flask, we seeded them and found that tumorspheres were able to adhere and acquire the same morphology than their corresponding adherent-cultured cells (Fig. 1f). Moreover, gene expression profiles showed no differences between adherent cells directly established from tissue and those established from tumorspheres, confirming that tumorspheres are able to adhere and differentiate, losing the expression of stemness markers reported in the next section.

### Lung tumorspheres overexpress genes related to stemness and invasion

**Fig. 2:**
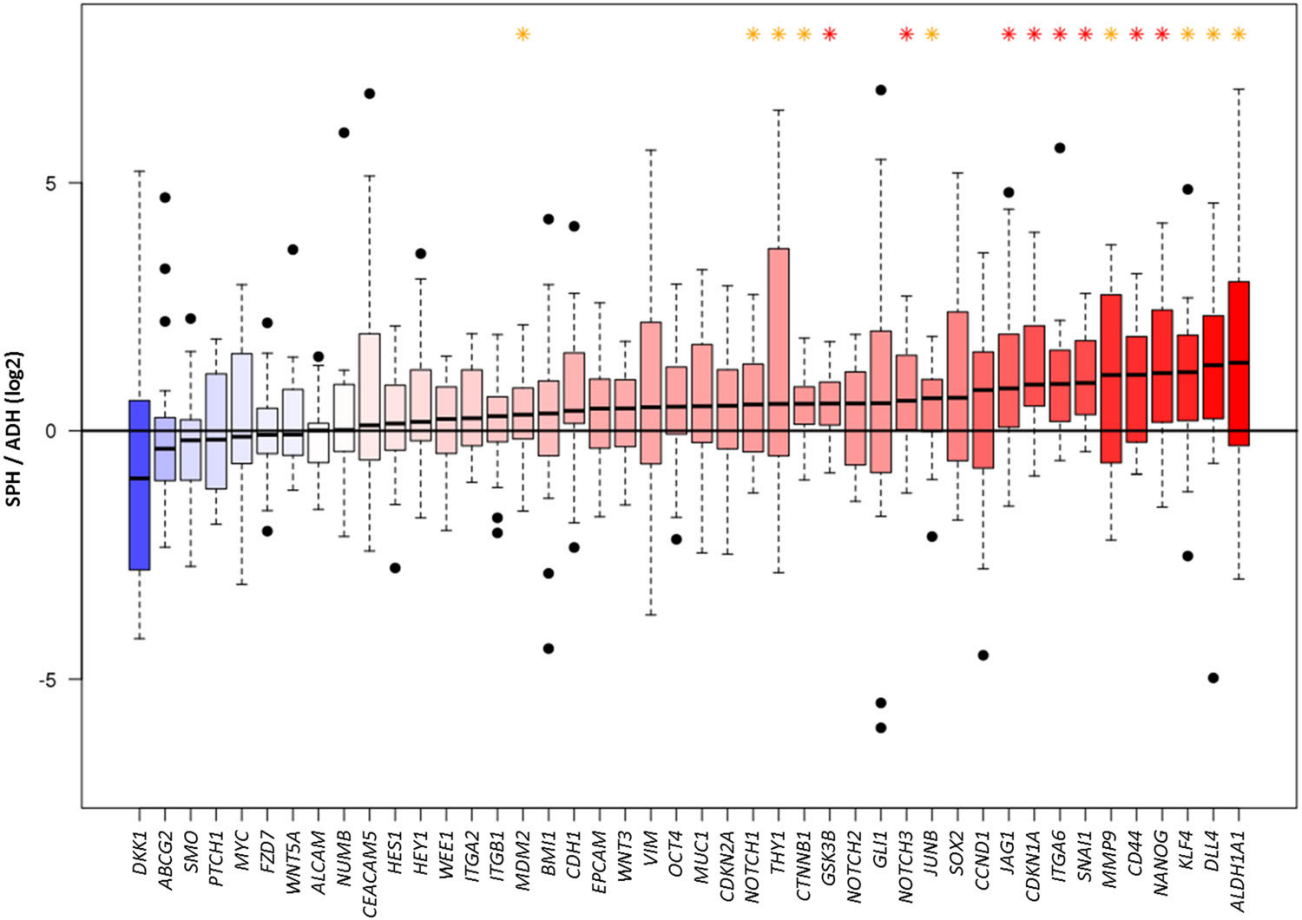
Transcription levels of CSC-related genes in tumorspheres vs. adherent-cultured cells. mRNA was measured by RTqPCR. The results shown are the log 2 of the ratio between the gene expression of tumorspheres and the gene expression of adherent-cultured cells. Error bars represent SEM. Asterisks indicate *p* < 0.05 (yellow) and *p* < 0.01 (red)

The expression at mRNA level of 51 genes described as potential lung CSC markers, pluripotency and cell cycle regulators, invasion promoters, and components of Notch, Wnt, and Hedgehog signaling pathways was analyzed in tumorspheres and adherent cells from patient-derived cells and cell lines using RTqPCR. The relative expression levels of *LIN28B*, *CD133*, *WNT1*, *WNT2*, *SHH*, and *GLI1* were below the limit of detection of the technique in most samples and were excluded from the final analysis. Remarkably, the expression of *CD133* could not be detected using three different sets of gene expression assays. Tumorspheres showed higher expression of 37 out of 44 genes compared to adherent-cultured cells, being a group of 17 genes: *ALDH1A1*, *KLF4*, *NANOG*, *CD44*, *THY1*, *CDKN1A*, *JUNB*, *MDM2*, *MMP9*, *SNAI1*, *ITGA6*, *NOTCH1*, *NOTCH3*, *DLL4*, *JAG1*, *CTNNB1*, and *GSK3B*, significantly overexpressed according to Wilcoxon’s signed-rank test (Fig. 2).

Unsupervised PCA including patient samples and cell lines was performed in order to group samples according to gene expression. PCA score plot revealed that the adherent-cultured cells population is more homogeneous than tumorspheres in terms of gene expression (Supplementary Fig. S5). Next, a supervised partial least square-discriminant analysis was applied to discriminate tumorspheres and adherent-cultured cells. As shown in Supplementary Fig. S6, principal component 1 (PC1) was able to separate most CSCs from adherent tumor cells. Loading plot revealed that the expression of *SNAI1*, *GSK3B*, *CD44*, *CDKN1A*, *NOTCH3*, *NANOG*, and *CTNNB1* genes in tumorspheres contributed the most to this separation. To analyze the differences between cell lines and patient-derived cultures in their gene expression profile, PCA analyses were applied separately to cell lines and patient-derived cultures. The PCA score plot from cell lines exhibited the high variability between them. PC1 separated 7 out of 12 tumorspheres cultures from adherent cells (Supplementary Fig. S7). On the contrary, in the PCA score plot corresponding to the patient-derived cultures, PC1 separated FIS343 and FIS320 from the rest of primary cultures, whereas PC2 clearly distinguished adherent tumor cells from lung tumorspheres (Supplementary Fig. S8). When comparing the gene expression profiles, it was observed that expression of *BMI1*, *CD166*, *CDKN2A*, *MDM2*, *HEY1*, *NUMB*, *ITGA6*, and *NOTCH3* was induced in tumorspheres from FIS343 and FIS320 patients, while tumorspheres from the rest of patients showed higher expression of *EPCAM*, *NOTCH1*, *NOTCH2*, *CD44*, *CTNNB1*, *MMP9*, and *CDKN1A*. Thereafter, a logistic regression model was used to reduce dimensionality and remove collinear expression of genes. The model was built considering the 17 statistically significant overexpressed genes (*p* < 0.05, Wilcoxon’s signed-rank test), being *CDKN1A*, *NOTCH3*, *CD44*, *ITGA6*, *NANOG*, and *SNAI1* the genes selected for further analyses.

### Lung adenocarcinoma tumorspheres overexpress p21, Notch3, CD44, integrin α6, Nanog, and Snail

**Fig. 3:**
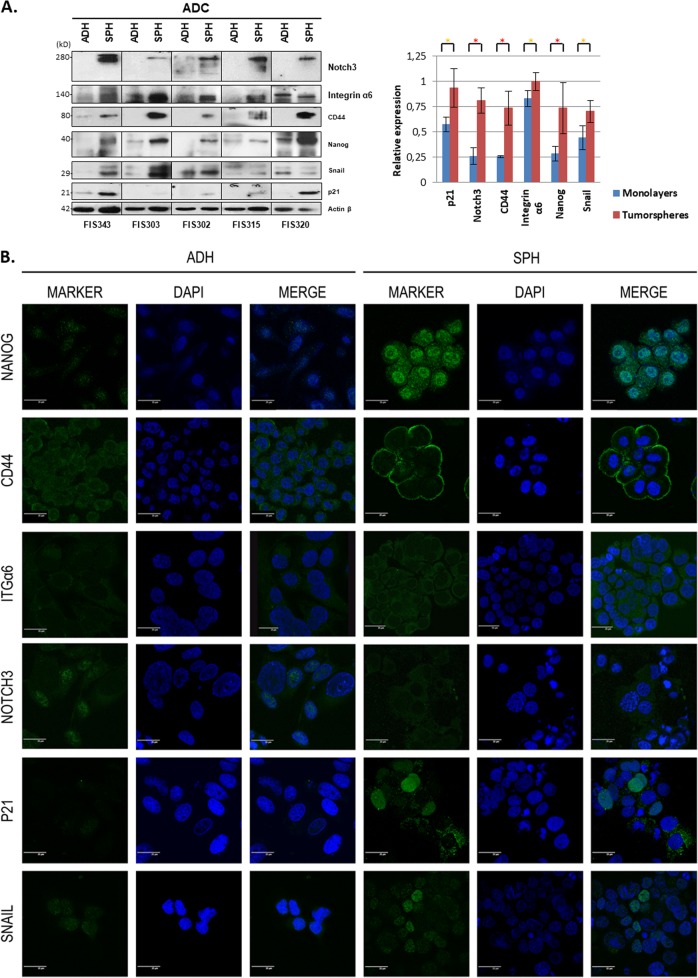
Analysis of CSC-related proteins in primary cultures. Representative immunoblots (**a**) and immunofluorescence (**b**) images of Nanog, CD44, integrin α6, Notch3, p21, and Snail in adherent-cultured cells and tumorspheres from ADC patients. Bar chart represents the relative expression of each protein according to immunoblots. Asterisks indicate *p* < 0.05 (yellow) and *p* < 0.01 (red). β-Actin was used to assess equal loading in immunoblots. Green channel in immunofluorescence shows the indicated antibody staining, blue channel shows DAPI staining, and merge shows all channels merged. Scale bar represents 25 µm

Gene expression analyses were complemented with immunoblotting (IB) and immunofluorescence (IF) analyses of the proteins encoded by these six genes in primary cultures. All protein expressions were significantly higher in tumorspheres than in adherent cells in lung ADC patients according to IB and only one patient (FIS320) showed higher levels of integrin α6 and Snail in the adherent-cultured cells than in tumorspheres (Fig. 3a). Interestingly, IF showed differential subcellular localization of p21, which was nuclear and cytoplasmic in cells forming lung tumorspheres and only nuclear in adherent tumor cells (Fig. 3b, secondary antibody control Supplementary Fig. S9). For Notch3, membrane localization in addition to cytoplasmic and nuclear was detected in both adherent cells and lung tumorspheres. In addition, all cells forming tumorspheres expressed both CD44 and Nanog, although signals showed polarity, being higher on cell membranes of cells located in the periphery of the lung tumorspheres for CD44 and nuclear for Nanog. In contrast, the expression of Nanog and CD44 was notably lower in adherent cells. Integrin α6 showed similar expression pattern to CD44 with higher expression in cytoplasm, especially in the cell membrane. Finally, Snail was overexpressed in tumorspheres and showed a non-uniform nuclear location along them. In adherent cells, a more homogeneous expression was detected in nuclei, which was weaker than that observed on their corresponding tumorspheres.

In contrast to ADC patients, variability was greater for SCC patients (Supplementary Fig. S10). No significant differences were found for p21, CD44 and integrin α6, whereas higher expression of Nanog, Snail, and Notch3 were detected in adherent cells in this histology, suggesting that different molecular changes govern CSCs in this tumor subtype. Complementary analyses of other potential CSC-related proteins (CD133, CD166, ALDH1A1, β-catenin, E-cadherin, and vimentin) and histological markers (carcinoembryonic antigen, cytokeratin 7, p63, cytokeratins 5/6, CD56) were performed and can be found in the Supplementary Figs. S11 and S12.

### A CSCs score is a prognostic biomarker for OS in NSCLC

Data from TCGA for ADC and SCC patients were used to associate genes significantly overexpressed in tumorspheres with survival. Clinicopathological characteristics of these patients are summarized in Table 2 (in silico set). Patients with post-surgical complications were excluded from the survival analysis, and only those patients who had at least 1 month of follow-up were included (*N* = 661). Cox regression and Kaplan–Meier analyses indicated that patients with high levels of *THY1*, *SNAI1*, *ITGA6*, and *CDKN1A* presented worse OS (Supplementary Table S4 and Supplementary Fig. S13). Survival analyses were also performed according to patient histology, associating high *ITGA6* and *JAG1* with worse prognosis in ADC patients. No other significant associations were found between survival and clinicopathological variables or gene expression.

**Table 2:** Clinicopathological characteristics of the patients included in the study

	In silico set				Validation set	
	Global cohort		ADC cohort		ADC cohort	
	*N* = 661	%	*N* = 345	%	*N* = 114	%
Age at surgery (median, range)	68 [38–88]		67 [38–88]		65 [37–84]	
Gender						
Male	395	59.8	165	47.8	77	67.5
Female	266	40.2	180	52.2	37	32.5
Stage						
I	375	56.7	197	57.1	73	64.0
II	179	27.1	90	26.1	26	22.8
IIIA	107	16.2	58	16.8	15	13.2
Histology						
ADC	345	52.2	345	100.0	114	100.0
SCC	316	47.8	0	0.0	0	0.0
Others	0	0	0	0	0	0.0
Performance status						
0	NS		NS		79	69.3
1					35	30.7
Differentiation grade						
Poor	NS		NS		17	14.9
Moderate					38	33.3
Well					27	23.7
NS					32	28.1
Smoking status						
Current	165	25.0	83	24.1	52	45.6
Former	382	57.8	179	51.9	39	34.2
Never	114	17.2	83	24.1	23	20.2
Exitus						
No	400	60.5	231	67.0	65	57.0
Yes	261	39.5	114	33.0	49	43.0

*ADC* adenocarcinoma, *NS* non-specified

**Fig. 4:**
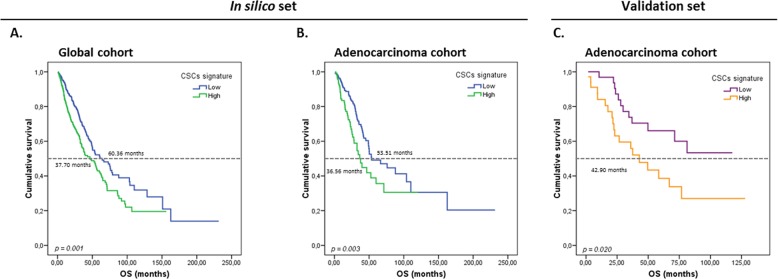
Prognostic value of the CSC score. Kaplan–Meier plots for OS according to the CSC score in the entire cohort (**a**) and the adenocarcinoma subcohort (**b**) from TCGA and the validation cohort (**c**). The signature was divided as low and high according to its median. Green and orange lines represent patients with high levels of expression, whereas blue and purple lines represent patients with low levels of expression. *P* values were calculated using the Kaplan–Meier test

Thereafter, we intended to create a gene expression score that can provide more accurate predictions for patients’ prognostic^27,28^. Univariate Cox regression analysis was performed considering OS as a dependent variable. Genes were ordered on the basis of their prognostic power (univariate *Z*-score, Supplementary Fig. S14), and according to this ranking, the expression of *CDKN1A*, *SNAI1*, and *ITGA6* were found to be associated with survival (*Z*-score >1.5), and therefore were selected to create a risk signature. We constructed a model based on the relative contribution of these three genes in the multivariate analysis (considering absolute regression coefficients, see Supplementary Table S5), and the resulting score was named CSCs score, with the following equation: (*CDKN1A*x0.123) + (*ITGA6*x0.196) + (*SNAI1*x0.255). Kaplan–Meier analysis showed that patients with high CSC score (>median) had shorter OS (37.7 vs. 60.4 mo, *p* = 0.001; Fig. 4a). We also performed a stratified analysis by histology and found a similar association between high CSC score and prognosis for ADC patients (36.6 vs. 53.5 mo, *p* = 0.003; Fig. 4b). To evaluate the potential use of the CSC score as an independent prognostic biomarker, a multivariate analysis was performed including all the significant variables from the univariate analyses (age, tumor node metastasis (TNM) staging, tumor size, lymph node involvement, *CDKN1A*, *ITGA6*, *SNAI1*, and the CSC score). Results obtained from this multivariate analysis indicated that age, TNM staging, and the CSC score in the entire cohort and TNM staging, lymph node involvement, and the CSC score in the ADC cohort were independently associated with survival (see Table 3).

**Table 3:** Results from the multivariate Cox regression model for OS

	Global cohort (*N* = 661)			ADC cohort (*N* = 345)		
	HR	95% CI	*P* value	HR	95% CI	*P* value
Age (>65 vs. ≤65)	1.398	1.069–1.827	0.014	–	–	–
TNM staging (IIIA vs. II vs. I)	1.353	1.161–1.578	0.0001	1.515	1.097–2.092	0.012
LN involvement (yes vs. no)	–	–	–	2.108	1.453–3.059	<0.0001
CSC signature (high vs. low)	1.498	1.167–1.922	0.001	1.869	1.275–2.738	0.001

*ADC* adenocarcinoma, *LN* involvement, lymph node involvement, *OS* overall survival, *HR* hazard ratio, *CI* confidence interval

An independent cohort of patients with resected lung ADC was used for validation of the CSC signature. Clinicopathological characteristics of these patients are summarized in Table 2 (validation set). Cox regression and Kaplan–Meier analyses of individual genes indicated that patients with high expression levels of *SNAI1* and *ITGA6* presented worse OS (Supplementary Table S4 and Supplementary Fig. S13). In addition, the association between high CSC score and worse prognosis was confirmed in this cohort (42.90 vs. not reached (NR) mo, *p* = 0.020; Fig. 4c).

## Discussion

Cancers exist in an extraordinary variety of types and subtypes, making each cancer individually unique. Tumors are heterogeneous and many cancer cell populations with different features are present. Among these tumor populations, cells with stemness properties, commonly called CSCs, have been described and associated to more aggressive phenotypes. There is strong evidence suggesting that cancer cells with stem properties selectively resist current cancer therapies, indicating the important role that CSCs play in tumor evolution, relapse, and metastasis^8,9^. In this context, evaluation of their gene expression profiles could provide the basis for identifying biomarkers and therapeutic targets, which could improve patients’ outcomes. Sphere-forming assays are well-described culture methods that have been used for stem cells isolation, identification, and enrichment from different tissues^10–14^. Starting material for these cultures can also be commercial cell lines, but using cells directly isolated from surgical resections specimens reflect in vivo conditions better than they do, since immortalized cell lines do not behave as primary cultures and long-term manipulation alters phenotype, functions, and responsiveness to stimuli. In addition, clinicopathological information from primary cultures can be correlated with each culture behavior, whereas this information is limited for most cell lines. As a result, although the establishment of primary cultures can be problematic and time-consuming, experiments with this type of approach are required to strengthen the findings, especially when studying stemness properties. In addition, the expression of some surface markers has been proposed as characteristic of lung CSCs, although contradictory data can be found between different studies, even when using the same cell lines^15–18^.

For these reasons, in this study we used unsorted primary tumor cells to form tumorspheres without selecting them according to the expression of a single marker or combination that could misrepresent the cell population with stem properties. Under these conditions, the success rate in the establishment of primary cultures from lung cancer patients was 40%, which is in consonance with results previously reported^12,29^. Although *EGFR*, *ALK*, and *ROS1* are common driver mutations in lung adenocarcinoma with effective targeted therapies approved for use^30,31^, we found no correlations between their mutational status, *KRAS* or *TP53* mutational status, or other clinicopathological variables and the establishment of primary cultures.

To ensure that our lung tumorspheres were enriched in cells with stem-like properties, multiple analyses were performed to determine if they have the fundamental features of this population. Patient-derived tumorspheres had self-renewal and unlimited exponential growth abilities, higher tumorigenic potential in vivo than adherent control cells, were able to differentiate and acquire the properties of their adherent counterparts, showed high invasion capacity, and were very resistant to high doses of chemotherapeutic agents. These results are in line with those previously reported on CSCs isolated from lung cancer^16–18^ and confirm that this approach can be used for their enrichment in a simple and cost-effective way. In addition to all these properties, CSCs share with other stem populations the overexpression of stemness pathways, cytoprotective enzymes, and efflux pumps^32–34^. Different genes and molecules have been proposed as characteristic of lung CSCs, but most studies are focused on few cell lines and analyzed the expression of small groups of genes, being very challenging to determine which ones are characteristic of a particular cell line or patient and which ones are governing stemness in lung CSCs^33–37^.

In this study, lung tumorspheres exhibited increased expression of genes encoding for cytoprotective enzymes (*ALDH1A1*), pluripotency inducers (*KLF4*, *NANOG*), cell cycle regulators (*CDKN1A*, *JUNB*, *MDM2*), metastasis-related genes (*CD44*, *THY1*, *MMP9*, *SNAI1*, *ITGA6*), and components of Notch (*NOTCH1*, *NOTCH3*, *DLL4*, *JAG1*), and Wnt (*CTNNB1*, *GSK3B*) pathways. To reduce the dimensionality of our data, a mathematical algorithm was used, selecting *CDKN1A*, *NOTCH3*, *CD44*, *ITGA6*, *NANOG*, and *SNAI1* to distinguish tumorspheres from adherent tumor cells. Protein analyses of the selected genes confirmed gene expression results and showed that proteins encoded by these genes are overexpressed in tumorspheres from ADC patients and in some cases differentially located along the cells. These molecules are widely related to features observed in CSCs and could constitute potential targets. For instance, cytoplasmic p21 (encoded by *CDKN1A*) and Notch3 were associated with self-renewal, tumorigenic behavior, and aggressive tumors^38–41^. Additionally, Snail has been reported to regulate Nanog, inducing stemness properties in lung cancer and, along with integrin α6, Snail, and CD44, has been associated with cell migration, invasion, and metastasis^42–46^. In contrast, variability was greater for SCC patients, for whom no significant results were found for p21, CD44, and integrin α6, whereas the expression of Notch3, Nanog, and Snail seemed higher in adherent cells than in tumorspheres. Differences in the role of these molecules have been previously reported according to tumor histology^47,48^, highlighting that there are significant changes between NSCLC subtypes and that markers to identify CSCs are not only tissue-dependent but also histological. In this study, only one 1 of the 12 cell lines and 3 out of the 8 primary cultures were lung SCC, so a greater number of cases could be required to properly identify markers and targets for this tumor subtype. Complementary, the protein expression of other molecules was analyzed, finding differential expression of CD133, CD166, ALDH1A1, β-catenin, E-cadherin, and vimentin in patients’ tumorspheres. Our group previously reported the association of some these molecules to CSCs, being their expression associated to the response to compounds targeting cell stemness^49^.

Many studies have tried to correlate the expression of genes associated with CSCs to patients’ prognosis^50,51^. Nevertheless, most of them are focused on single pathway-specific markers with limited prognostic value. Finding gene expression signatures that identify altered pathways in carcinogenesis could lead to the discovery of molecular subclasses and predict patients’ outcomes better^52,53^. TCGA data of the genes significantly overexpressed in tumorspheres was ranked according to their prognostic power, trying to find a gene signature, which could provide valuable prognostic information^24,25^. We created a score combining the expression of *CDKN1A*, *ITGA6*, and *SNAI1*, which was an independent prognostic biomarker for lung cancer patients. To validate it, the expression of these genes was evaluated in an independent cohort of resected lung ADC patients, finding that patients with elevated CSC score had shorter OS. These results are of great importance because current clinicopathological staging methods have limited success in predicting patient survival and today we still cannot predict which patients will be cured, and which ones will relapse after surgery. Gene expression scores based on RTqPCR have demonstrated being useful for classifying tumors and predicting prognosis, being even approved as prognostic tools in clinical practice^54^. This technology is a well-implemented methodology in our group for biomarkers’ research, previously reporting angiogenesis and immune checkpoint scores for NSCLC^55,56^. The CSCs score proposed can help in future clinical practice, since high scores may reflect a bigger CSC population with enhanced cell migration, invasion, and tumor initiation capacity that will be able to modulate cell cycle and persist after cancer treatments. As a result, patients with high values on this score may need adjuvant treatment and should be closely followed after a successful surgery, because they have a higher risk to die. The development of targeted therapies against this tumor population is essential to prevent relapse of patients and improve their future outcome.

Our approach allows the establishment of long-term lung CSCs cultures, being a powerful tool for identifying the molecular alterations present in this tumor cell population and providing new insight into the field of CSCs in NSCLC. Tumorspheres can be used for CSC enrichment and a common set of genes were found to be significantly and consistently overexpressed in them. Developing new therapeutic strategies against these molecules could have major implications in patients’ survival. In addition, a gene score based on molecules overexpressed in this tumor cell population predicts worse outcome in two independent cohorts of patients, representing an independent prognostic biomarker that can be used to determine the outcome in resectable lung ADC patients.

## Supplementary information

Supplementary Fig. S1

Supplementary Fig. S2

Supplementary Fig. S3

Supplementary Fig. S4

Supplementary Fig. S5

Supplementary Fig. S6

Supplementary Fig. S7

Supplementary Fig. S8

Supplementary Fig. S9

Supplementary Fig. S10

Supplementary Fig. S11

Supplementary Fig. S12

Supplementary Fig. S13

Supplementary Fig. S14

Supplementary Table S1

Supplementary Table S2

Supplementary Table S3

Supplementary Table S4

Supplementary Table S5

Supplementary Video 1
