# Supplementary figures and images for: Lung tumorspheres reveal cancer stem cell-like properties and a score with prognostic impact in resected non-small-cell lung cancer
Source: Cell Death Dis. 2019 Sep 10;10(9):660. doi: 10.1038/s41419-019-1898-1 (PMC6737160; doi:10.1038/s41419-019-1898-1)

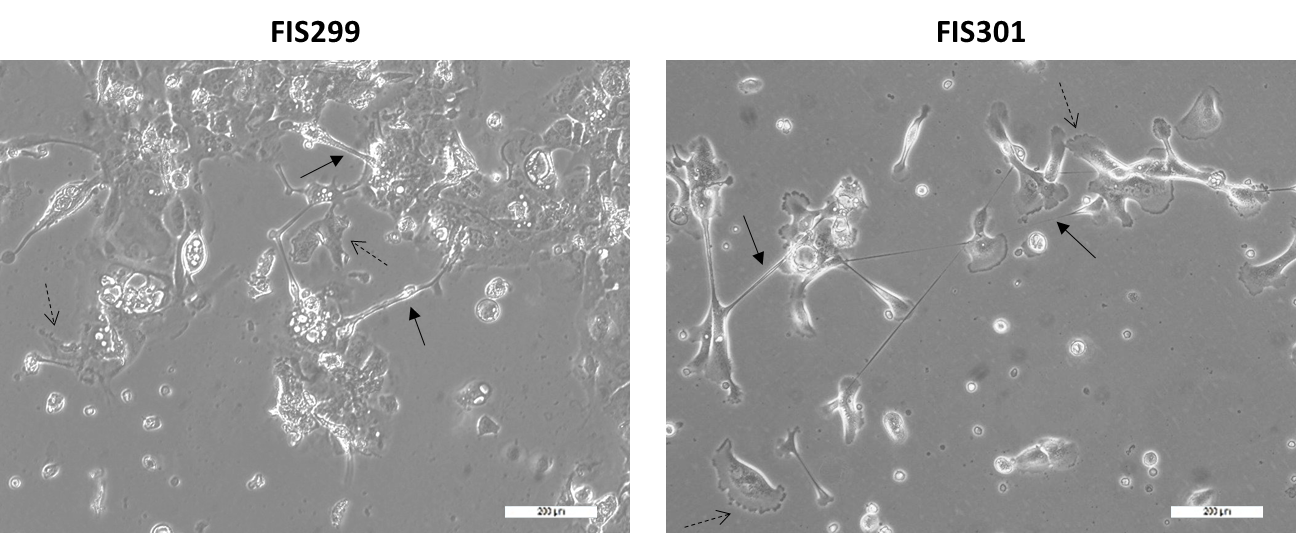

Supplement: Supplementary file 1 — Supplementary Fig. S1 [file 41419_2019_1898_MOESM1_ESM.tif]

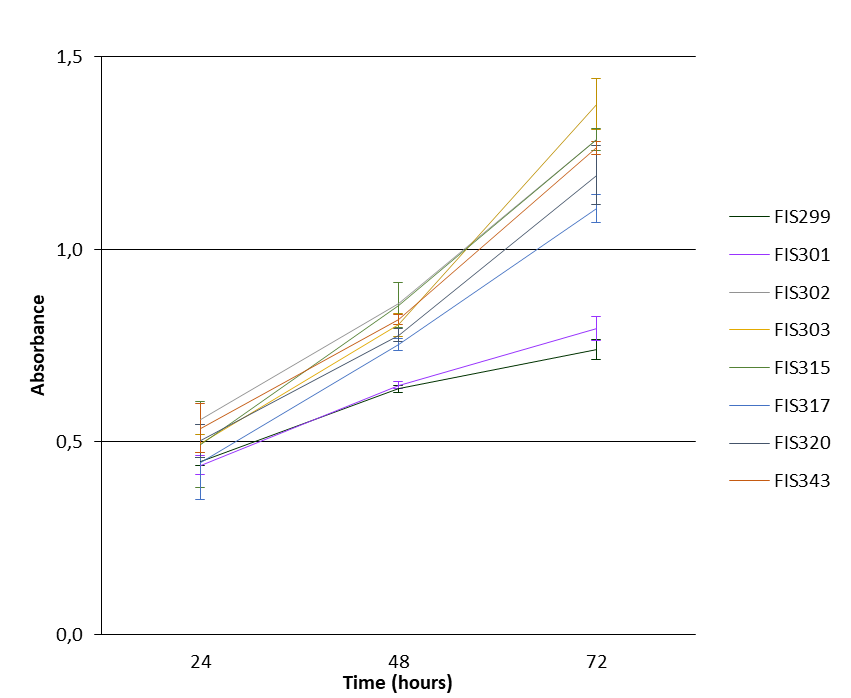

Supplement: Supplementary file 2 — Supplementary Fig. S2 [file 41419_2019_1898_MOESM2_ESM.tif]

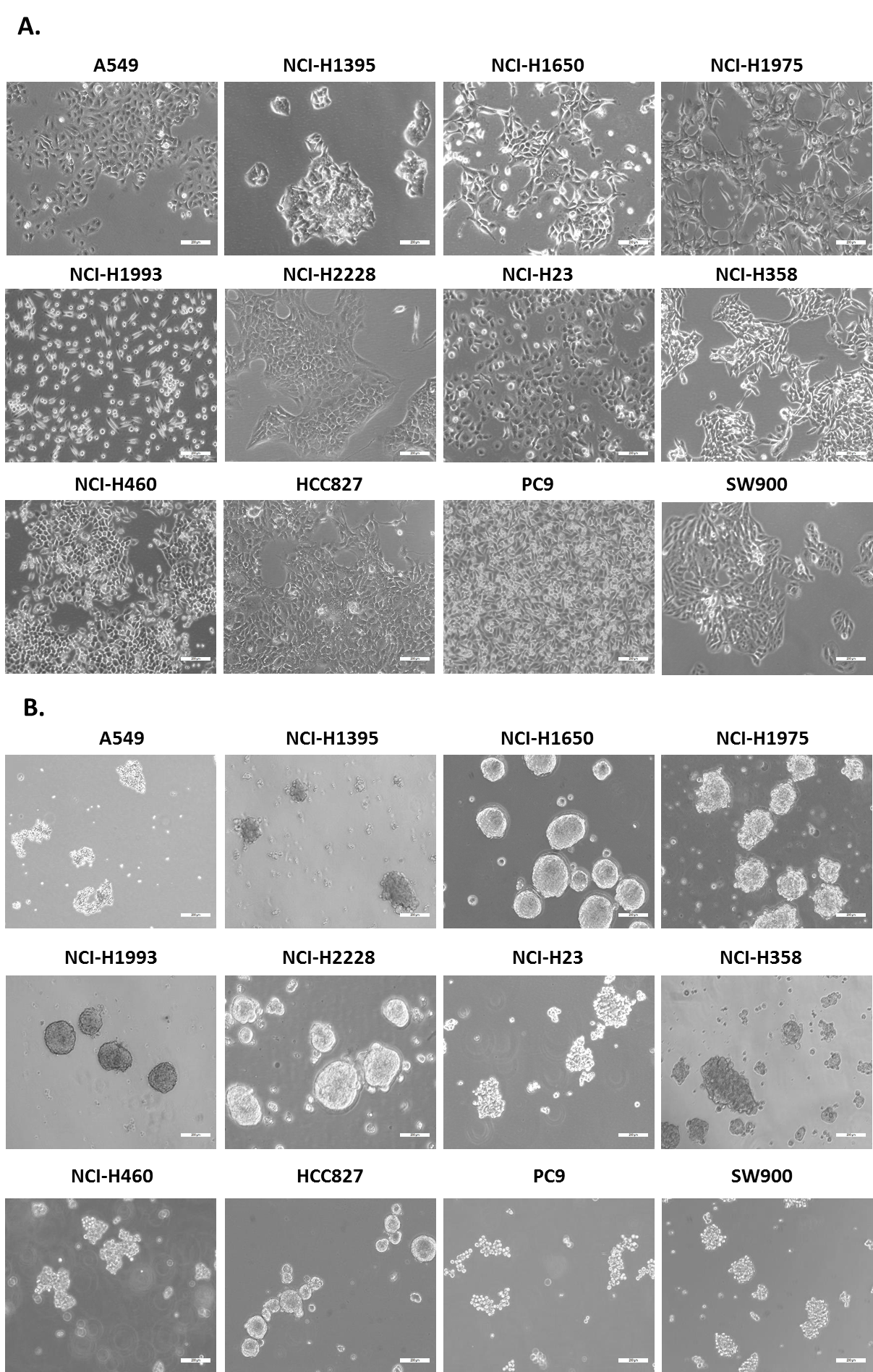

Supplement: Supplementary file 3 — Supplementary Fig. S3 [file 41419_2019_1898_MOESM3_ESM.tif]

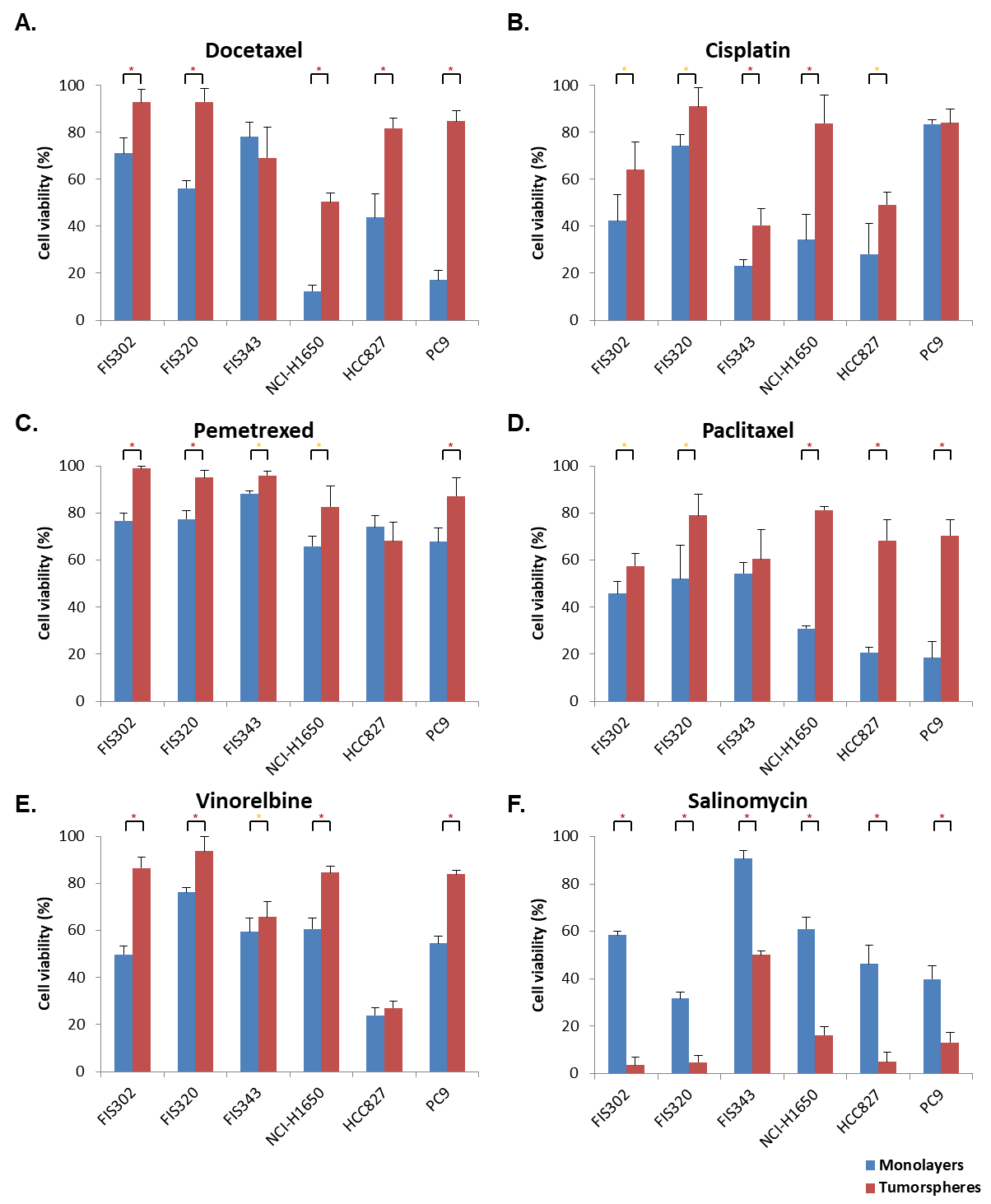

Supplement: Supplementary file 4 — Supplementary Fig. S4 [file 41419_2019_1898_MOESM4_ESM.tif]

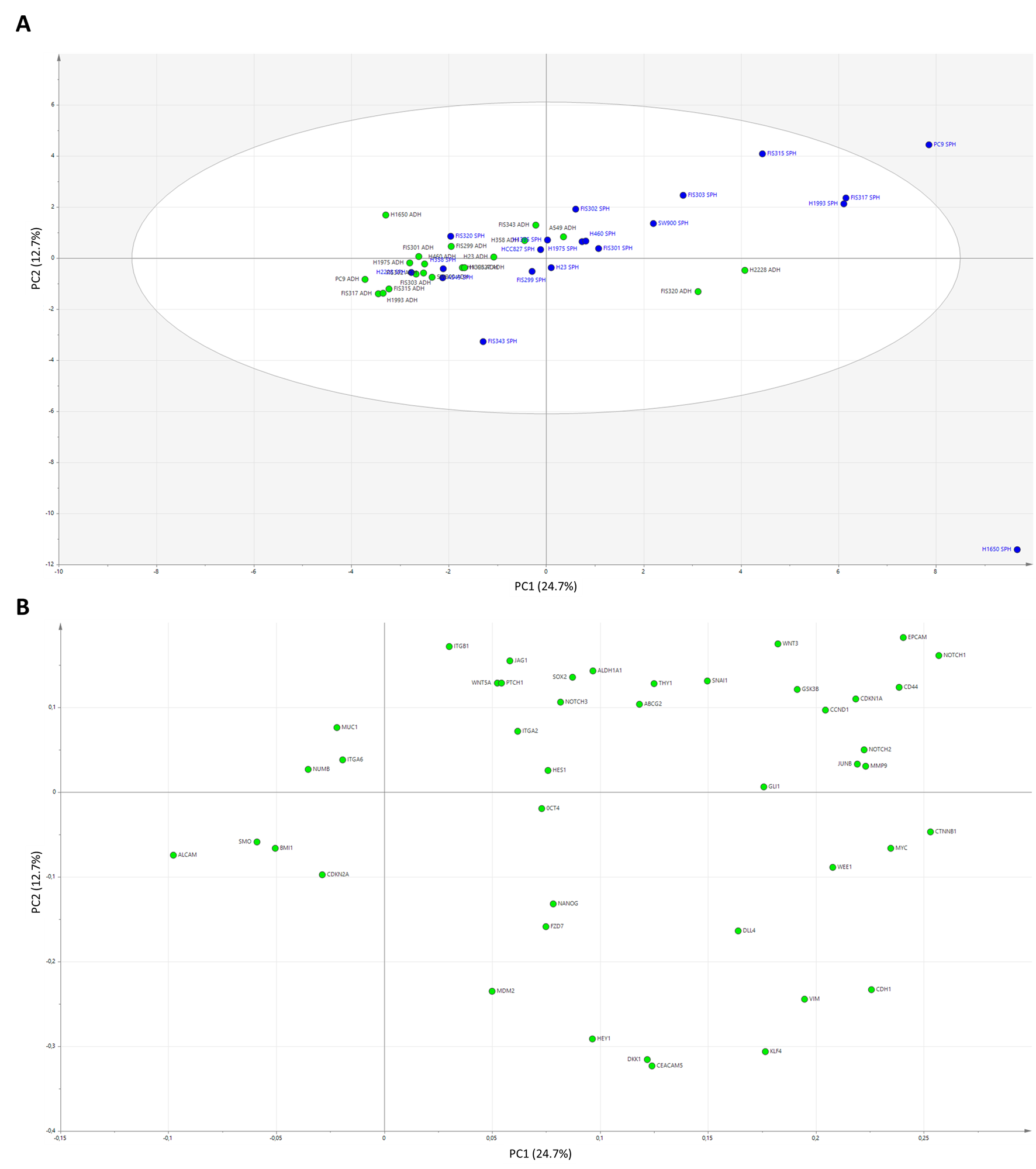

Supplement: Supplementary file 5 — Supplementary Fig. S5 [file 41419_2019_1898_MOESM5_ESM.tif]

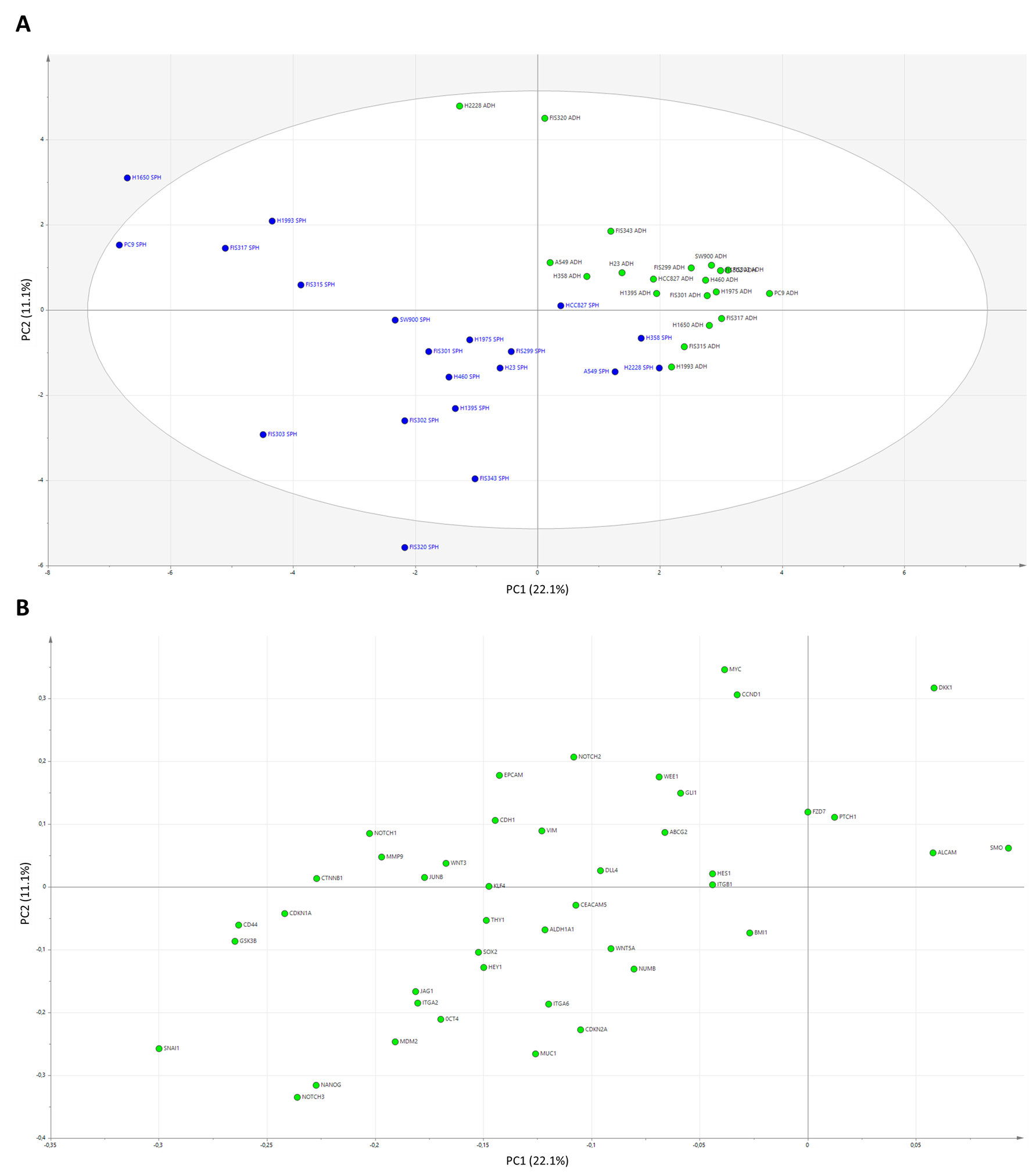

Supplement: Supplementary file 6 — Supplementary Fig. S6 [file 41419_2019_1898_MOESM6_ESM.tif]

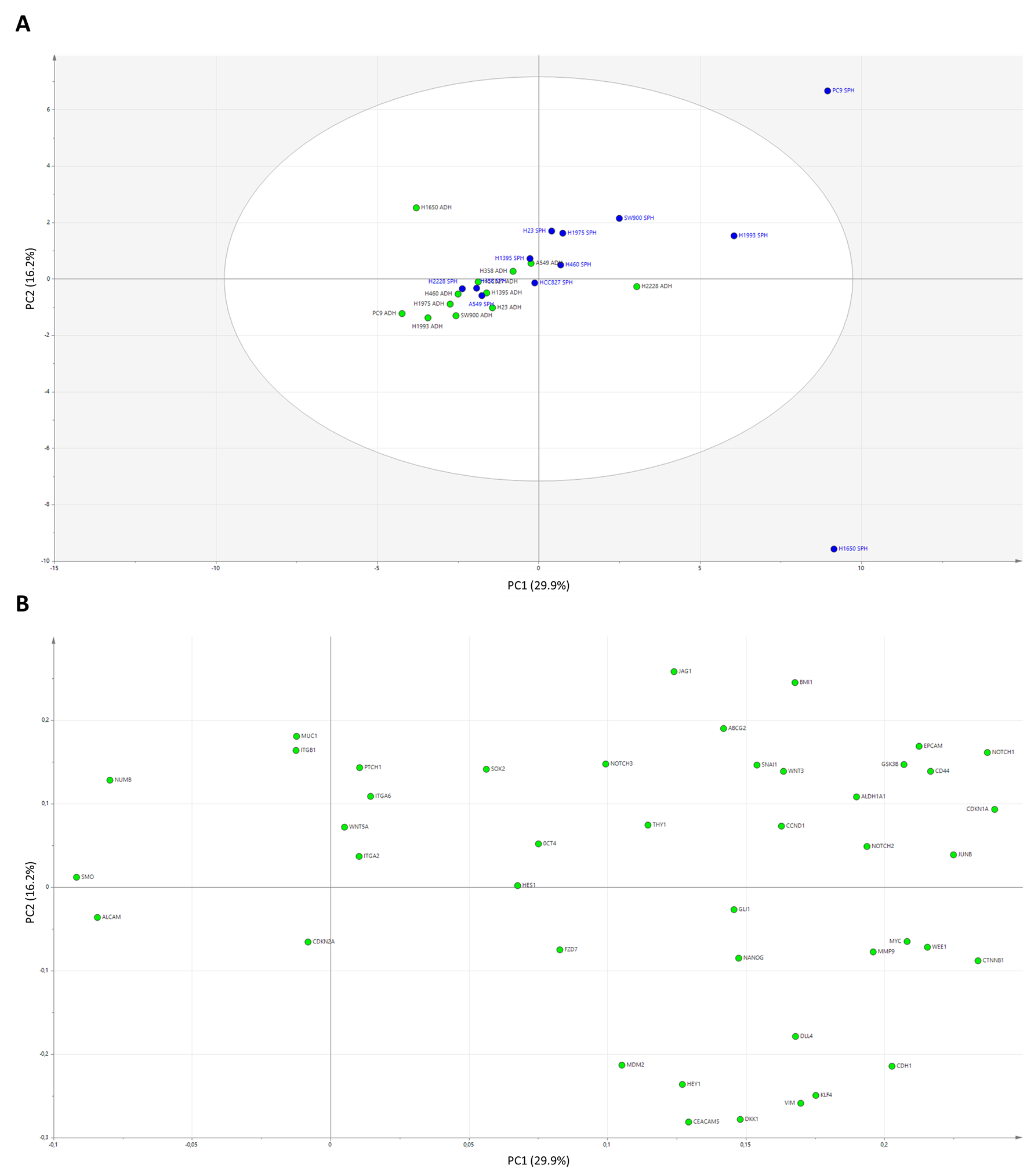

Supplement: Supplementary file 7 — Supplementary Fig. S7 [file 41419_2019_1898_MOESM7_ESM.tif]

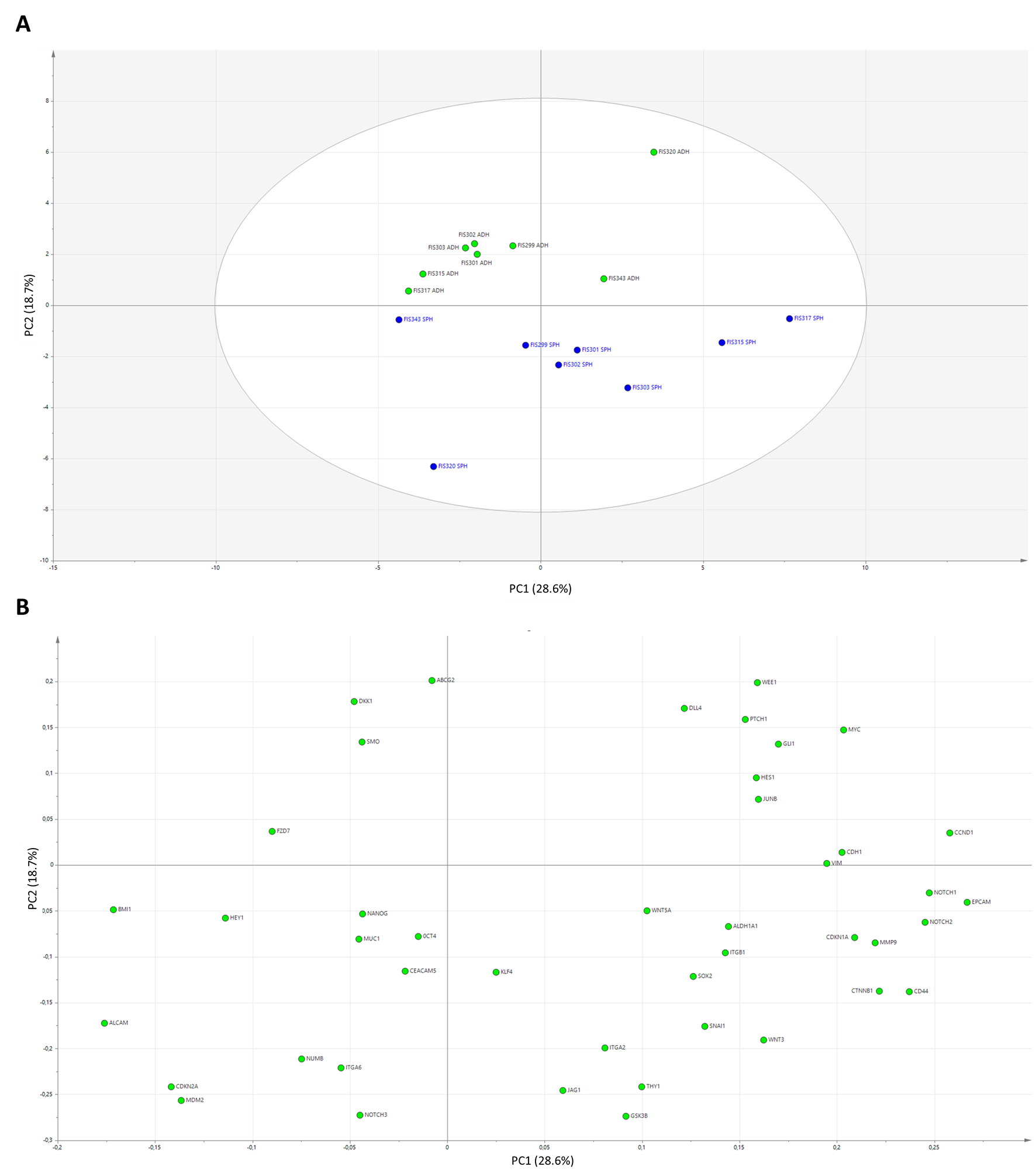

Supplement: Supplementary file 8 — Supplementary Fig. S8 [file 41419_2019_1898_MOESM8_ESM.tif]

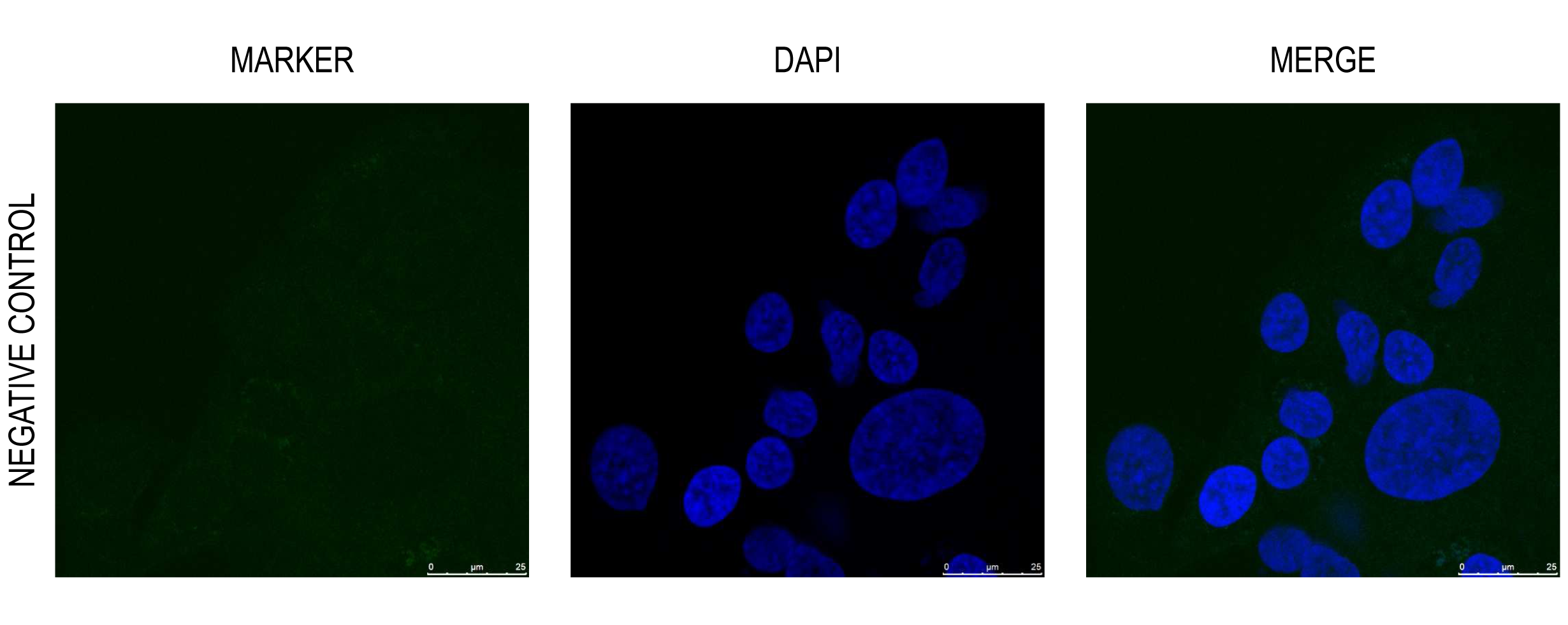

Supplement: Supplementary file 9 — Supplementary Fig. S9 [file 41419_2019_1898_MOESM9_ESM.tif]

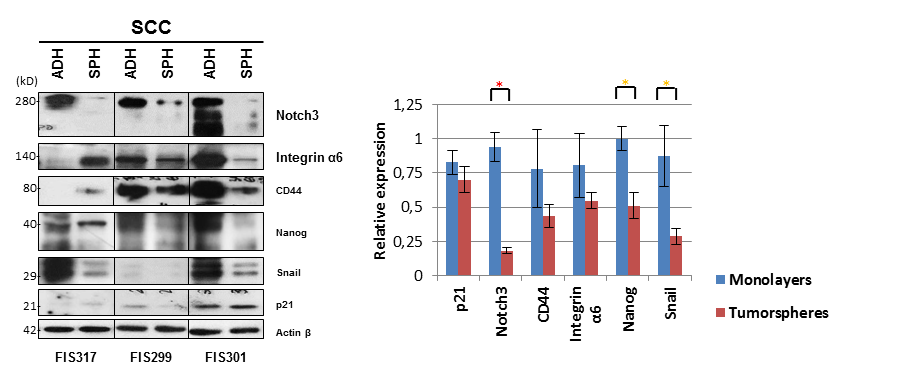

Supplement: Supplementary file 10 — Supplementary Fig. S10 [file 41419_2019_1898_MOESM10_ESM.tif]

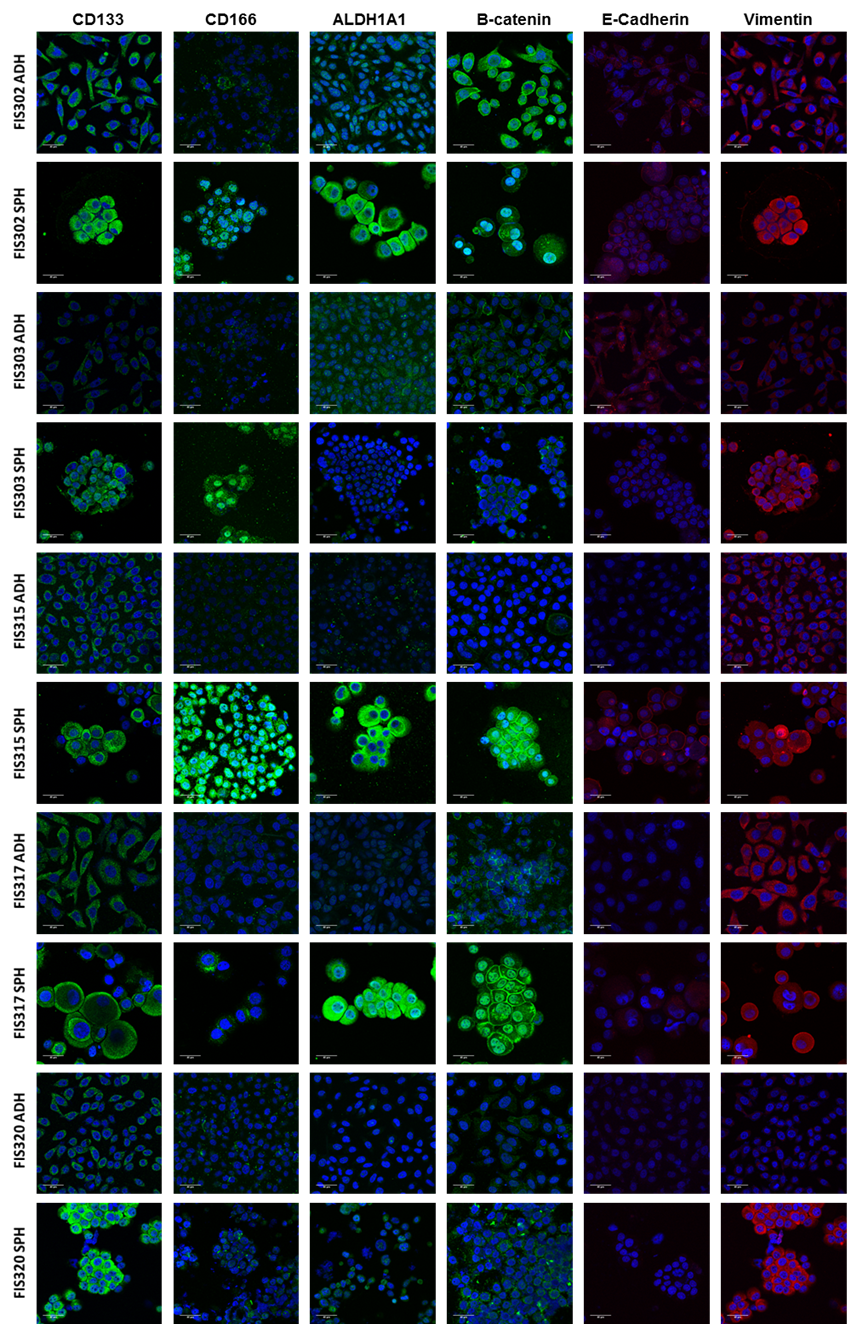

Supplement: Supplementary file 11 — Supplementary Fig. S11 [file 41419_2019_1898_MOESM11_ESM.tif]

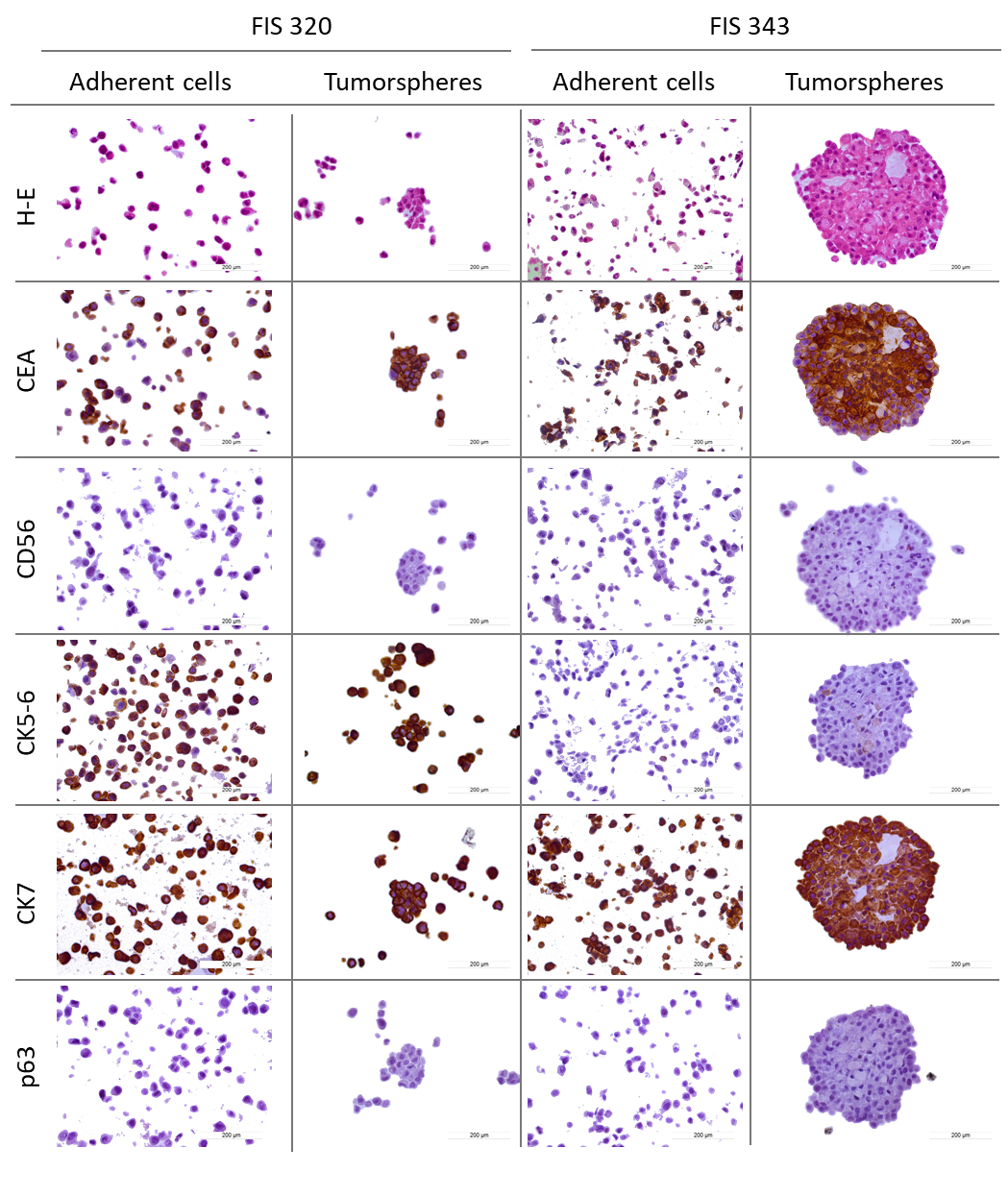

Supplement: Supplementary file 12 — Supplementary Fig. S12 [file 41419_2019_1898_MOESM12_ESM.tif]

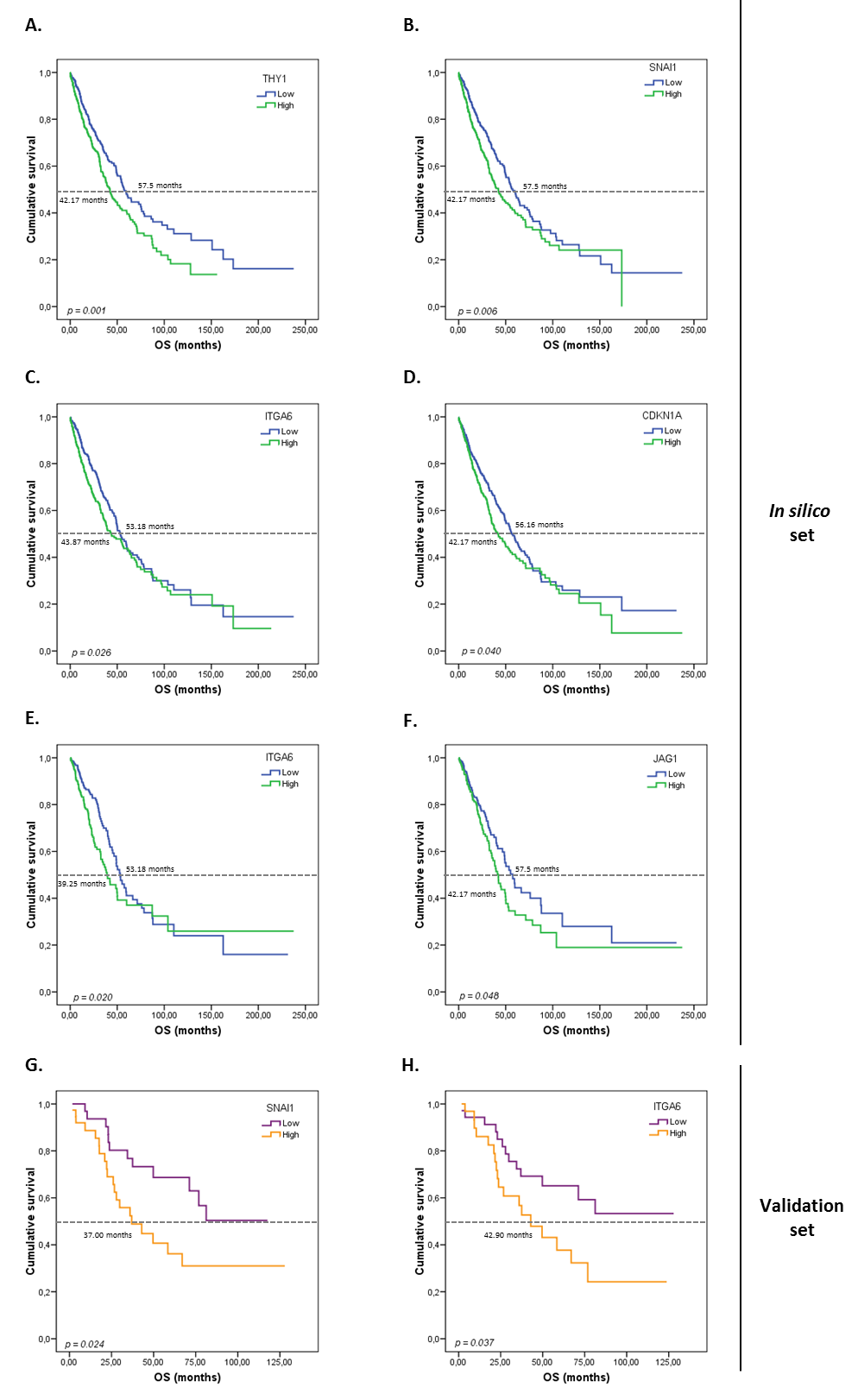

Supplement: Supplementary file 13 — Supplementary Fig. S13 [file 41419_2019_1898_MOESM13_ESM.tif]

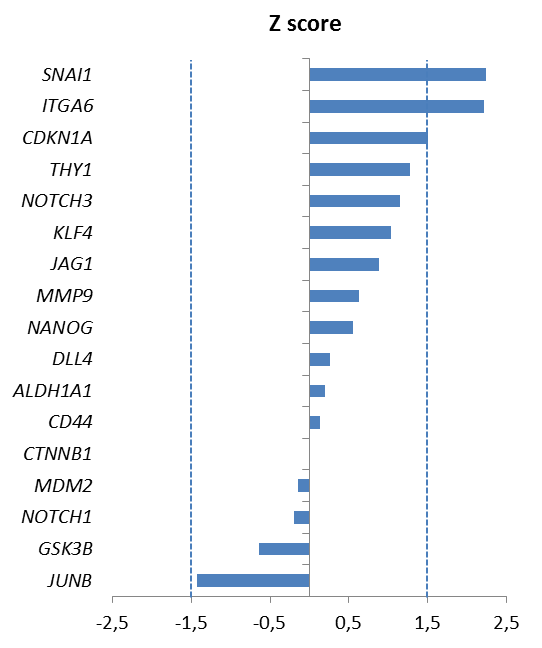

Supplement: Supplementary file 14 — Supplementary Fig. S14 [file 41419_2019_1898_MOESM14_ESM.tif]
